# A Powerful Strategy for Carbon Reduction: Recyclable Mono-Material Polyethylene Functional Film

**DOI:** 10.3390/polym16152196

**Published:** 2024-08-01

**Authors:** Liming Wei, Shengqi Zhan, Mingyu Zhou, Xuerong Xu, Feng You, Huaming Zheng

**Affiliations:** 1 Shanghai Royal New Materials Technology Company Limited, Shanghai 201803, China; baozhuang126@126.com; 2 Province Key Lab of Plasma Chemistry and Advanced Materials, Wuhan Institute of Technology, Wuhan 430073, China; shengqizhan1314@gmail.com (S.Z.); zmy1522785080@163.com (M.Z.); wxuerong@126.com (X.X.); youfeng.mse@wit.edu.cn (F.Y.)

**Keywords:** mono-material, multilayer, recycling, CO_2_ emissions

## Abstract

Given the abundant plastics produced globally, and the negative environmental impacts of disposable plastic products throughout their life cycle, there has been significant attention drawn by the general public and governments worldwide. Mono-material multilayer packaging is a potent strategy to address the challenge of carbon emissions as it offers specific functionalities (such as strength and barrier properties) through its layers and facilitates recycling. In this study, a five-layer co-extruded polyethylene composite film LLDPE/mPE/PVA/mPE/LLDPE was taken as a model to investigate its mechanical properties and barrier properties after four recycling cycles. The result revealed that the longitudinal tensile strength and transvers tensile were, respectively, dropped from 29.66 MPa and 24.9 MPa to 21.972 MPa and 19.222 MPa after the recycling; it is shown that the film still has good mechanical properties after the recycling cycle. However, a noticeable decline in the barrier properties was observed after the second recycling. In contrast to traditional plastics, a mono-material film with a 10 wt.% circulating mass could reduce CO_2_ emissions by 3692.25 kg for every 1.0 ton of plastic products after four recycling cycles.

## 1. Introduction

The plastic industry has a history of over 110 years, dating back to the first plastic product (1907). Since the 1950s, with the advancement of petrochemical technology, the global plastic industry has experienced rapid growth, reaching an annual output value of 4.6 × 10^8^ tons [1], and one of the main applications for plastics is packaging materials [2]. Moreover, plastic is known for its low density, excellent mechanical and optical properties, outstanding barrier properties, strong weather resistance, ease of processing, and affordability, and its widespread use in various sectors of society has greatly facilitated people’s lives [3].

However, a large amount of discarded plastic products are resistant to decomposition for a long period of time. Notably, the additives added during the production process pose severe threats to soil, water resources, and marine life [3]. Currently, the highly toxic and persistent organic pollutants (POPs) generated via municipal solid waste incineration technologies bring serious risks to human health and hinder social progress and sustainable economic development. In China, millions of tons of plastic packaging materials are utilized every year, with one-third of them failing to be effectively treated. Considering the substantial quantities of agricultural films and medical supplies, the overall amount of non-degradable plastics reaches up to 50 million tons per year, which directly leads to serious environmental pollution problems [4,5].

It has also been reported that one in three species of marine life have been found entangled in marine litter, and 90% of all seabirds have plastic in their stomachs [6]. Dr. Ian A. Kane’s group from the University of Manchester further highlighted that the accumulation of microplastics on the seabed is also alarming. In the southeastern waters of Corsica in the Mediterranean, there are approximately 1.9 million microplastic fragments per square meter of the seabed [7]. These microplastic components primarily consist of polyethylene (PE), polyethylene terephthalate (PET), and nylon (PA) [8]. In fact, numerous monomers and chemical additives are used during plastic processing, which pollutes the aquatic environment and ultimately disrupts the endocrine system of organisms. Considering the significant environmental harm caused by traditional plastics, the United Nations released a global review of national laws and regulations to restrict disposable plastics and microplastics in 2019 [9].

In light of the significant environmental challenges posed by traditional petroleum-based plastic materials, the scientific and industrial communities are actively engaged in the development and promotion of innovative recycling strategies. The physicochemical properties of plastics permit their classification into two categories: thermoplastics, which have favorable recyclability and can be reused through melt remolding, and thermoset plastics, which are less amenable to recycling due to their structural stability and difficulty in remolding after curing, rendering them more challenging to recycle. In order to address this challenge, four principal methods of plastic recycling have been widely discussed and implemented [10]. Closed-loop recycling involves the direct reprocessing of plastics through mechanical sorting and purification, resulting in products with properties comparable to those of the original materials. This approach effectively closes the loop on the recycling of plastics. Degradation recycling, on the other hand, involves processing plastics into materials or products with slightly inferior properties. Despite the decrease in quality, these materials can effectively extend the service life of the materials, making this a viable option for certain applications. Chemical recycling employs chemical means to break down plastics and recover monomers or other chemicals. This method is particularly suited to materials that are difficult to mechanically recycle. Energy recovery utilizes plastics as a fuel and converts them into energy through combustion or pyrolysis. While this is a last resort, it is an effective means of reducing the volume of waste in certain instances. In recent years, scholars have continued to investigate novel methods for advancing the frontiers of conventional recycling. To illustrate, Yue L. et al. transformed thermoset plastics into recyclable dynamic networks through a vitrification reaction using a special catalyst, thereby providing a novel solution to the recycling challenges of thermoset plastics [11]. Furthermore, Ghabezi P. et al. demonstrated the compositing of waste polypropylene with waste carbon fibers, which not only achieved high-value utilization of waste but also developed high-performance composites suitable for 3D printing [12]. This strategy fully embodies the concept of a circular economy. In this context, this study will focus on the pathway of mechanical recycling to investigate its effect on the properties of five-layer co-extruded film materials. Through the optimized mechanical recycling process, the physical properties of the film after reuse of the recycled material can be ensured, and the carbon footprint of the whole production cycle can be effectively reduced. This provides a scientific basis and practical guidance for achieving the green transformation and sustainable development goals of the plastics industry.

To address the environmental issues associated with traditional petroleum-based plastics, many countries around the world have been investing in the development and production of biodegradable plastic alternatives in recent years, including polybutylene adipate terephthalate (PBAT), polylactic acid (PLA), 1,4-Butanediol-succinic acid copolymer (PBS), polyhydroxyalkanoate (PHA), polypropylene carbonate (PPC), etc. Although these bioplastics can reduce the emission of carbon dioxide during their lifecycle, the economic cost is significantly higher than that of traditional plastics. Additionally, the bioplastics often lack barrier and weather resistance, which limits their widespread use. To overcome these challenges, a mono-material strategy that combines the advantages of traditional plastics and biodegradable materials has emerged as a promising strategy to facilitate the recycling of waste plastics. In response to growing concerns about plastic packaging waste, policies aimed at reducing plastic consumption and promoting recycling have been implemented globally. For instance, the European Commission’s “Plastic Strategy in the Circular Economy” aims to make all plastic packaging reusable or easily recyclable by 2030.

As is well known, polymer chains are known to be susceptible to breakage under the influence of thermal shear fields, leading to a decline in their performance after repeated shearing, which consequently affects the overall quality of the products. In this study, a five-layer polyethylene composite film consisting of LLDPE, mPE, PVA, mPE, and LLDPE layers was prepared via a multilayer co-extrusion technology. Meanwhile, the relationship between the number of recyclable times and the corresponding changes in the performance of the film was systematically investigated. Moreover, an in-depth comparison and analysis of the carbon emissions throughout the lifecycle of the co-extruded composite film was conducted, with the purpose of providing theoretical support for the global implementation of “circular economy” and “dual carbon economy” principles [13,14,15,16].

## 2. Materials and Methods 

### 2.1. Materials and Reagents

Experimental materials: low density polyethylene (LLDPE 7042, Sinopec), metallocene polyethylene (mPE 3518CB, ExxonMobil), polyvinyl alcohol (PVA 318, Colali, Japan), and grafted PE (sPE, Laboratory Made Specialty Grafts).

### 2.2. Equipment and Instruments

Experimental instruments: five layer co-extrusion blown film machine (PBSI-20/28, Guangzhou Putong Experimental Analysis Instrument Co., Ltd., Guangzhou, China), parallel twin-screw extruder (HK36/48, Nanjing Keya Chemical Equipment Co., Ltd., Nanjing, China), high-speed mixing machine (Z300/600, Zhangjiagang Wuhe Machinery Co., Ltd., Zhangjiagang, China), electronic universal testing machine (GBH-1, Guangzhou Standard Packaging Equipment Co., Ltd., Guangzhou, China), oxygen transmittance meter (Y210, Guangzhou Standard Packaging Equipment Co., Ltd.), water vapor transmittance meter (W413, Guangzhou Standard Packaging Equipment Co., Ltd.), and rotary rheometer (MCR-92, Anton Paar, Graz, Austria).

### 2.3. Method

#### 2.3.1. Experimental Sample Preparation

LLDPE, mPE, sPE, and PVA resins were blended uniformly in a high-speed mixer according to the formula set in Table 1. A multilayer co-extrusion blowing machine was used and the temperature ranges of each stage of the extruders were set at 200–220 °C to obtain an LLDPE/mPE/PVA/mPE/LLDPE five-layer co-extruded film (Fn). The films were crushed and granulated with a twin-screw extruder to obtain the recycled resins (HS-N). In every cycle, a part of LLDPE was replaced with HS-N at a fixed ration of 1:9.

The original mixture resins were blown, and the homologous films were denoted as F_0_.

The F_0_ was reused to obtain the resin which was denoted as HS-0. Additionally, 10 wt.% of LLDPE was replaced with HS-1. According to the formula in Table 1, the following blowing film LLDPE/mPE/PVA/mPE/LLDPE was denoted as F_1_ in the first cycle.

In the subsequent cycles, the recycled material and the five-layer co-extruded film are denoted as HS-0, HS-1, HS-2… HS-N and F_0_, F_1_, F_2_… Fn, respectively.

#### 2.3.2. Determination of the Molecular Weight

To analyze the molecular weight change in the polymers under the action of hot shearing, LLDPE/mPE/PVA/mPE/LLDPE resin was repeatedly extruded several times according to the formula in Table 1, and the resin after each extrusion was marked as HS-N. The molecular weight of HS-0, HS-1, HS-2, and HS-3 was detected with gel permeation chromatography GPC (PL-GPC50). The eluent was trichlorobenzene (TCB) containing 0.0125% dibutyl hydroxy toluene (BHT) using the column of PL gel MIXED-BLS 300 mm × 7.5 mm × 2 μm, and the related parameters were listed as follows: a flow rate of 1.0 mL/min, a temperature of 150 °C, and an injection volume of 200.0 μL. The number average molecular weight, weight average molecular weight, viscosity average molecular weight, and molecular weight distribution of four materials were analyzed during the recycling cycle, which was used to determine whether they could participate in the next round of recycling.

#### 2.3.3. Rheological Measurements

Rheological measurements were conducted with a rotary rheometer (MCR-92, Anton Paar) equipped with a parallel plates geometry and a circulating system for temperature control. Four types of resins (HS-0, HS-1, HS-2, and HS-3) were employed to determine the changes in viscosity. The samples were pressed into circular thin sheets (thickness of 1 mm and diameter of 25 mm) and the testing temperature was set at 190 °C. The mode of small amplitude oscillation shear was employed with an angular frequency ranging from 0.1 to 100 rad/s and a strain amplitude of 1%. The logarithmic point sampling method was used to collect data and determine the viscosity of materials at different cycling stages [17]. Based on the changes in material viscosity and zero-shear viscosity, the recycled resins were evaluated for their feasibility in the next round of recycling. All measurements were performed in duplicate.

#### 2.3.4. Mechanical Properties of Films

The mechanical properties of the recycled films, such as tensile strength (*TS*) and elongation at break (*EAB*), were measured using a universal testing machine (Instron 5565, Boston, MA, USA) according to ISO572-2,1993 (*E*). The rectangular strips with dimensions of 150 mm × 15 mm were tested using a span length of 50 mm and a stretching rate of 100 mm/min. Prior to the test, all samples were conditioned at 23 °C and 50% relative humidity (RH) for 24 h. The tensile strength and elongation at break were determined for each sample at least ten times. The tensile strength (*TS*) and elongation at break (*EAB*) were calculated according to Equations (1) and (2), respectively [18].

(1) $$TS=\frac{F}{15\cdot d}$$

where *TS* is the tensile strength of the film, MPa; *F* is the maximum tensile force experienced by the film during the stretching process, N; and *d* is the thickness of the film (mm).

(2) $$E=\frac{L-L_{0}}{L_{0}}\times 100\%$$

where *E* is the elongation at break of the thin film, %; *L* is the length at the time of film fracture, mm; and *L*_0_ is the initial distance between the two fixtures, mm.

#### 2.3.5. Barrier Performance of Water Vapor

The water vapor barrier properties of the recycled films were measured using a water vapor permeability analyzer (W405L, Guangzhou Biaoji Packaging Equipment Co., Ltd., Guangzhou, China). Before testing, the film should be equilibrated for 24 h in an environment of 23 °C and 65% RH relative humidity. The average value of four tests for each group of samples should be measured in g/(m^2^ • 24 h) [19].

#### 2.3.6. Barrier Performance of Oxygen

The oxygen barrier properties of the recycled films were conducted with the oxygen permeability analyzer (N530, Guangzhou Biaoji Packaging Equipment Co., Ltd.). Before testing, the thin film should be equilibrated for 24 h in an environment of 21 °C and 55% RH relative humidity. The test gas should be oxygen, and each group should be tested in triplicate, with an average value in cm^3^/m^2^• 24 h • 0.1 MPa) [20,21,22].

#### 2.3.7. Life Cycle Carbon Emission Analysis

**Figure 1:**
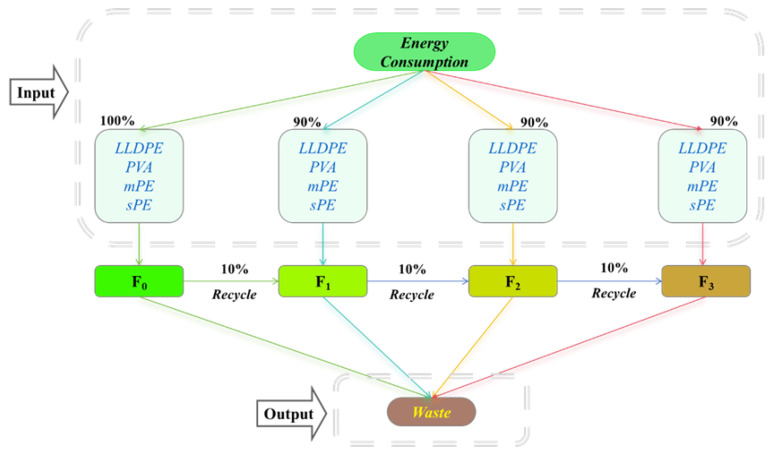
Diagram of the life cycle system of the five-layer co-extruded film.

The life cycle production system of the five-layer co-extruded film was quantified using a “cradle to grave” approach and a “top-down” model, in which a functional unit was established to produce 3 kg of the film. The system boundary included a linear system and cyclic system (Figure 1), and the analysis includes all life cycle stages from the raw material for the five-layer co-extruded film to its disposal.

For the recycling system, the analysis started with the production of the original plastic particles, and after the F_0_ stage, the waste of the five-layer co-extruded film was collected and a portion was selected for recycling, and the recycling film entered the next production–recycling cycle. The life cycle production system of the five-layer co-extruded film was composed of Input and Output. The Input included input raw materials and electricity, including the circulation system, and the final Output delivers the five-layer co-extruded film with raw materials, energy, emissions, and scrap in the production process. The circular production process was shown in Figure 1. The final result was analyzed with Openlca (https://www.openlca.org/) and Gabits (https://www.gabit.com/) software for life cycle analysis to explore the carbon emission generated in the production process [23,24].

## 3. Results

### 3.1. Molecular Weight Changes in the Recycled Films

It is well known that polymers with high molecular weight are prone to thermal degradation under the action of a thermal field accompanied by external shear, resulting in a decrease in molecular weight. When the molecular weight of the polymer decreases to a certain extent, the mechanical properties of the film will be significantly compromised [25]. After four recycling cycles, the molecular weight of the five-layer co-extruded film was altered as demonstrated in Table 2. On the whole, the number average molecular weight (Mn), weight average molecular weight (Mw), and viscosity average molecular weight (Mv) gradually decreased after recovery, while the changes in the molecular weight distribution (PD) were not significant. After the first recycling, the molecular weight of the film slightly decreased by ~0.17–5.5%. After the second recycling, the molecular weight of the film was reduced by ~20%. However, a significant reduction in molecular weight was not observed after the 3rd recycling. These results indicated that the molecular weight of the five-layer co-extruded film was thermally degraded along with the processing. On this basis, 10% of the recovery was fixed to maintain its ideal mechanical properties in the next cycle [26].

### 3.2. Analysis of Rotational Viscosity of Five-Layer Co-Extruded Film

**Figure 2:**
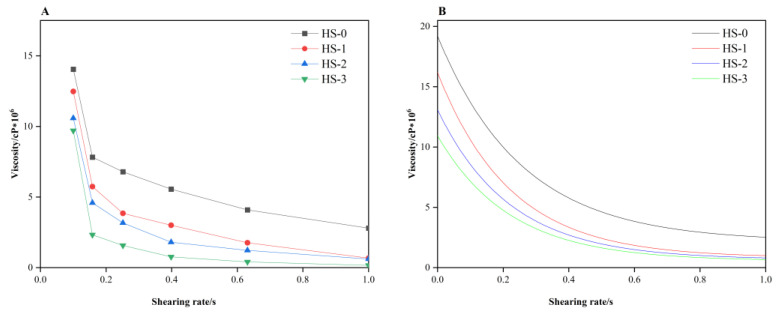
The shear viscosity of the five-layer co-extruded film after 100% recovery ((**A**) the viscosity changes in the blend composition; (**B**) the zero-shear viscosity of the samples).

Figure 2A illustrates the viscosity changes in the blend composition at 190 °C for different rotor speeds [27]. At a strain rate of 1.0%, the viscosity of the recycled resins (HS-0, HS-1, HS-2, and HS-3) was decreased with an increase in the angular frequency. Notably, the viscosity exhibited a sharp drop in the low-frequency region, demonstrating a shear thinning behavior of a pseudoplastic fluid [28]. Additionally, the viscosity values tended to decrease as the number of cycles increased [29]. After fitting and correcting the Carreau/Yasuda model, the zero-shear viscosity of the samples was calculated as shown in Figure 2B. The zero-shear viscosities of the four materials were not significantly different. For the same type of material, the zero-shear viscosity can reflect the magnitude of its average molecular weight. Generally, a low zero-shear viscosity indicates a small average molecular weight, while high zero-shear viscosity indicates a large average molecular weight. Therefore, the average molecular weight could be ranked as HS-0 > HS-1 > HS-2 > HS-3 [30].

### 3.3. Mechanical Performance Analysis

**Figure 3:**
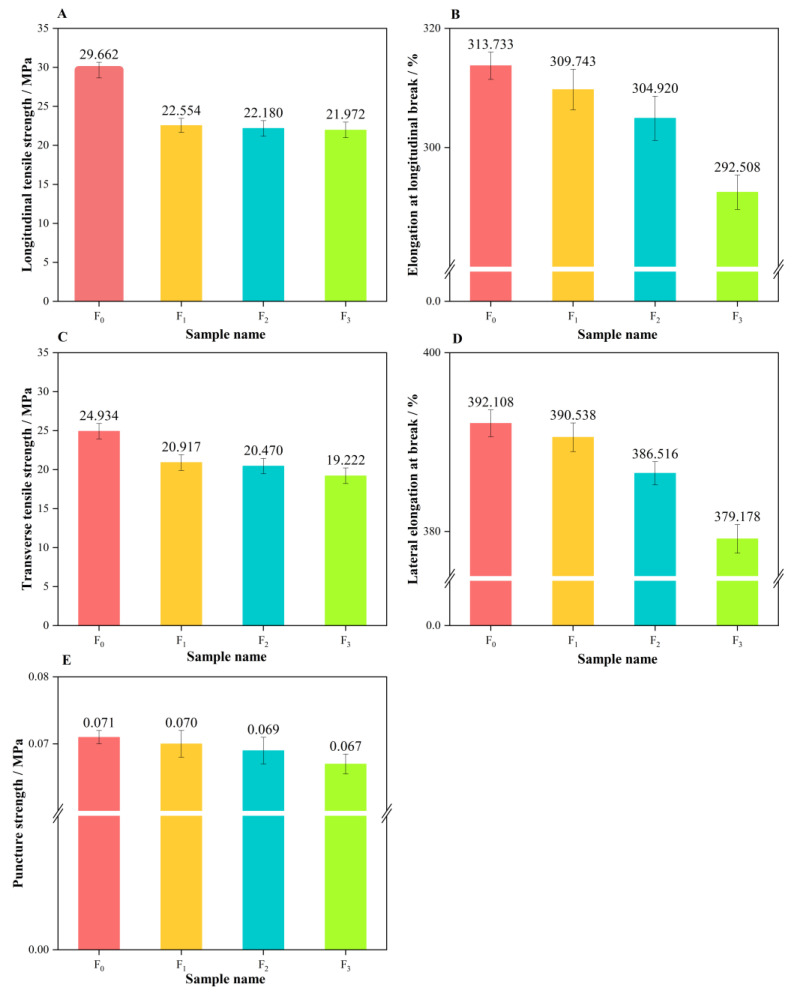
The mechanical properties of the recycled films. ((**A**) is the longitudinal tensile strength comparison of the recycled films; (**B**) is the comparison of elongation at longitudinal break of the recycled films; (**C**) is the comparison of transverse tensile strength of the recycled films; (**D**) is the comparison of transverse elongation at break of the recycled films; (**E**) is the comparison of puncture intensity of the recycled films).

Figure 3 illustrates the effects of recycling times on the mechanical properties of the recycled films [31,32]. The *TS* (tensile strength) of the recycled films was decreased with the increase in recycling times [26]. After the first recycling, the transverse tensile strength and longitudinal tensile strength decreased from 24.9 MPa and 29.66 MPa to 20.9 MPa and 22.55 MPa, respectively. After the second and third recycling, there was no significant change in the *TS*. During the subsequent recycling processes, the *EAB* (elongation at break) value was slightly increased. The puncture strength (PS) of the recycled films remained at 0.07 MPa. However, when the number of recycling times exceeded four, there was a round decrease in both of *TS* and PS owing to severe thermal degradation of the polymer resins [33,34,35].

### 3.4. Water Vapor and Oxygen Permeability

In Table 3, both the water vapor transmittance and oxygen transmittance increased to a certain extent with the increase in cycling times [36]. After the first cycling, the permeability of water vapor was increased from 7.559 to 9.305 g/(m^2^•24 h), and the permeability of oxygen was increased from 8.5371 to 10.4033 cm^3^/(m^2^•24 h•0.1 MPa). However, the film’s barrier property deteriorated after the second cycling. It is widely acknowledged that the barrier properties of polymers are closely linked to their surface polarity and internal structure. After many instances of shearing at high temperatures, the polymers underwent thermal oxidative degradation, resulting in a reduction in molecular weight and crystallinity, as well as carboxylation of the resin. Water vapor and carboxylated resin had similar polarity characteristics, which explained the significant increase in the water vapor transmittance of the film after the second cycling [37,38].Oxygen, being a non-polar molecule, exhibited repulsion with weakly polar resins, but the substantial decrease in the film’s crystallinity allowed for substantial permeability of oxygen within its internal structure, thereby contributing to a certain degree of increase in oxygen permeability [33,35,39].

### 3.5. Life Cycle Carbon Emission Analysis of Five-Layer Co-Extruded Recycled Film

To simplify the cycle process analysis of life cycle analysis, 10% recycled materials were incorporated into the system instead of using LLDPE for the next cycle. The recycled material proportion in the first cycle is 0.6% According to the system boundary principle (less than 1% of raw materials can not participate in the calculation), the calculation can be ignored, greatly simplifying the inventory analysis. Introducing the life cycle into the experiment enables the exploration of how the production process of a five-layer co-extruded film with 10% recycled raw material for recycling can reduce CO_2_ emissions within the boundaries of the experiment. The scope boundary of the system is from the preparation of LLDPE from petroleum to the scrapping of five-layer co-extruded thin films (after four recycling cycles), in which less than 1% of the contribution will be cut out. The system list is shown in Table 4 below.

The waste list of the production process within the system boundary range obtained from the recycling cycle of the five-layer co-extruded film can be seen in Table 5 below.

In the waste list generated by the system boundary of the five-layer co-extruded film, greenhouse gas CO_2_ is the largest waste unit, and the advantages and disadvantages of two non-production methods are compared with the emission of CO_2_ [6,14,15,23]. Under the measure of 10% material recovery (four cycles), the production of 4.0 kg five-layer co-extruded film will produce 10.72 kg of greenhouse gas CO_2_. The production of 4.0 kg of five-layer co-extruded films in a linear production mode without a recovery cycle generates 14.42 kg of greenhouse gas CO_2_. For every 1.0 kg five-layer co-extruded film produced via 10% raw material recovery/recycling, the emission of CO_2_ will be reduced by 3.69 kg. In the case of industrial mass production, the emission of CO_2_ will be reduced by 3692.25 kg for every 1.0 ton of five-layer co-extruded film. This proves that recycling and utilizing the mono-material film is a powerful strategy for the goal of reducing carbon emissions.

## 4. Discussion

The objective of this study is to examine the alterations in characteristics and prospective contribution to environmental sustainability via the fabrication of five-layer co-extruded films (LLDPE/mPE/PVA/mPE/LLDPE) through the recycling of a fixed percentage (10%) of the waste material. By employing a range of characterization techniques and life cycle analysis methods, this study offers a comprehensive examination of the influence of the recycling cycle on film properties and carbon emissions. This provides a scientific foundation for the optimization of plastic recycling.

Molecular weight and shear viscosity characterization: the results demonstrate that the molecular weight of the film exhibits a declining trend with the intensification of thermal shear. However, with the increase in the number of recycling cycles, the reduction in molecular weight becomes less pronounced, and the molecular weight distribution remains relatively stable. Concurrently, the shear viscosity of the regenerated resin exhibits a notable decline in the low-frequency region, aligning with the pseudoplastic fluid characteristics. The accumulation of cycles further diminishes the viscosity, mirroring the reduction in molecular weight. This simultaneously creates conducive conditions for processing technology.

Mechanical properties: the tensile strength and elongation at break of the films exhibited a notable decline following the initial cycle. However, the rate of decline in these mechanical properties slowed down in subsequent cycles, suggesting the presence of a plateau period during which performance reduction occurs at a constant rate. This implies that, at a fixed percentage of recycling, the performance of the films can reach a stable level despite the decrease.

Barrier property analysis: the water vapor transmission rate and oxygen transmission rate increase with the number of cycles, indicating that recycling has a detrimental impact on the barrier property of the film. This finding highlights the necessity for future material design and formulation adjustments to enhance the barrier property of the film.

Life cycle evaluation: the results of the life cycle analysis demonstrate that the use of 10 wt.% recycled material for four cycles can reduce CO_2_ emissions by 3692.25 kg per 1.0 ton of plastic product, in comparison to the traditional linear film production process. This evidence substantiates the positive impact of the recycling strategy in reducing the carbon footprint.

In the face of the global challenge of reducing carbon emissions, a mono material is an effective measure to achieve material recycling. In order to meet certain properties of products, the number of recycling cycles is a main indicator in the production process. Studying the effects of the number of recycling cycles of a mono material provides a powerful strategy for the implementation of carbon reduction targets. In future research, recycling of other materials (PP, PET, PVC, etc.) needs to be further investigated.

## Figures and Tables

**Table 1:** Preparation list of five-layer of co-extruded film.

Extruder	Materials	Materials Proportion	Processing Temperature/°C
Co-extrud. 1	80%LLDPE + 20%mPE	25%	200/210/210/200
Co-extrud. 2	50%sPE + 50%LLDPE	20%	200/210/210/200
Main extrud.	100%PVA	10%	210/220/220/210
Co-extrud. 3	50%sPE + 50%LLDPE	20%	200/210/210/200
Co-extrud. 4	80%LLDPE + 20%mPE	25%	200/210/210/200

**Table 2:** Molecular weight change in the five-layer co-extruded film.

Samples	Mn	Mw	Mv	PD
HS-0	39,283	117,525	102,057	2.99
HS-1	37,765	111,010	96,895	2.94
HS-2	30,513	88,466	76,936	2.90
HS-3	30,144	84,980	74,571	2.82

**Table 3:** Water vapor and oxygen permeability of the cycled films.

Samples	WVP/g/(m^2^•24 h)	OP/cm^3^/(m^2^•24 h•0.1 MPa)
F_0_	7.5589	8.5371
F_1_	9.3053	10.4033
F_2_	118.1608	27.9727
F_3_	134.6395	47.1480

**Table 4:** Input and output of a five-layer co-extruded film life cycle system.

INPUT	Unit	Result
LLDPE	kg	0.6
PVA	kg	0.1
sPE	kg	0.2
mPE	kg	0.1
Energy	MJ	28.8
OUTPUT	Unit	Result
Five layers of co-extruded film	kg	1
Other		

Note: Life cycle inventory and impact assessment were performed using GaBi Education and open LCA 2.0.4. All datasets were taken from the GaBi ts Education database 2020 and GaBi 2016 database.

**Table 5:** Waste list for a five-layer co-extruded film recovery/cycle life cycle system.

Impact Category	Unit	Result
Carbon dioxide	kg	10.72475
Dust (unspecified, from stack)	kg	0.000137
Nitrogen oxides	kg	0.006652
Sulfur dioxide	kg	0.000775
Ethane	kg	0.071280

Note: Life cycle inventory and impact assessment were performed using GaBi Education and open LCA 2.0.4. All datasets were taken from the GaBi ts Education database 2020 and GaBi 2016 database.

## Data Availability

The data presented in this study are available on request from the corresponding author.
